# Takotsubo Syndrome in 2025: Evolving Concepts in Pathophysiology, Diagnosis, and Long-Term Management

**DOI:** 10.3390/jcm15010197

**Published:** 2025-12-26

**Authors:** Alyssa McKenzie, Raed Bargout

**Affiliations:** 1 School of Medicine, St. Georges University, West Indies 11739, Grenada; 2 Dignity Glendale Memorial Hospital, Glendale, CA 91204, USA

**Keywords:** Takotsubo syndrome, stress cardiomyopathy, broken heart syndrome, catecholamines, microvascular dysfunction, brain–heart axis, heart failure

## Abstract

Takotsubo syndrome (TTS) is an acute condition involving left ventricular dysfunction that may present clinically as acute coronary syndrome without obstructive coronary disease or congestive heart failure. Initially considered benign, TTS is now recognized as a complex neurocardiac disorder with hospital morbidity rates comparable to those of myocardial infarction, as well as similar long-term risks. Recent evidence establishes TTS as a multifactorial process involving catecholamine overload, coronary microvascular dysfunction, myocardial energetic abnormalities, and dysregulation of the brain and heart axes. Developments in echocardiography, cardiac magnetic resonance imaging, and improvements in diagnostic criteria have enhanced the recognition of syndromic phenotypes. Management of TTS continues to remain primarily supportive; however, recent studies have revealed improved functional outcomes with structured cardiac rehabilitation and cognitive behavioral therapies as the first long-term disease-altering approaches. Future studies should combine neurocardiology, imaging, and therapy-focused research. This review integrates the understanding of the epidemiology, pathophysiology, clinical features, diagnostic work-up, and management of TTS, with particular emphasis on developments emerging from the past decade.

## 1. Introduction

Takotsubo syndrome (TTS), also referred to as takotsubo cardiomyopathy, stress cardiomyopathy, or “broken heart syndrome,” is an acute and typically reversible form of left ventricular systolic dysfunction that clinically mimics acute coronary syndrome in the absence of obstructive coronary artery disease [1,2,3]. First described in Japan in 1990, the condition derives its name from the characteristic apical ballooning appearance resembling a *takotsubo*, a traditional octopus trap [4,5].

Initially regarded as a benign event, TTS is now identified as an acute heart failure syndrome associated with increased chronic hospitalization and mortality rates that are almost equivalent to those associated with myocardial infarction [1,2,6,7]. Large international registries, including the International Takotsubo (InterTAK) Registry and the Heart Failure Association (HFA) of the European Society of Cardiology, have described the epidemiology, diagnostic criteria, and heterogeneous clinical presentations [2,3,6,8].

Advances over the past decade in cardiac imaging, neurocardiology, and coronary physiology have expanded current understanding of TTS to be a multifactorial disorder involving complex interactions. Despite normalization of left ventricular ejection fraction, accumulating evidence suggests many individuals experience persistent symptoms, subclinical dysfunction, and increased long-term risk, challenging the traditional concept of complete recovery [9,10,11,12]. Neuroimaging studies further implicate alterations within central autonomic networks, supporting a role for brain–heart axes in both vulnerability and recovery [13,14,15,16].

Although numerous reviews have addressed individual aspects of TTS, many foundational publications predate recent developments in mechanistic phenytopying, advanced imaging, and long-term outcome studies. Consequently, the literature lacks an integrated synthesis linking evolving pathophysiologic concepts with diagnostic strategies, risk stratification, and management. This review therefore aims to provide an updated overview of TTS, with particular emphasis on advances emerging over the past decade that inform diagnosis, prognosis and long-term management.

## 2. Definitions, Nomenclature, and Classification

### 2.1. Historical Background

Takotsubo syndrome (TTS) was first identified in Japan in 1990 as transient left ventricular systolic dysfunction with a characteristic apical ballooning pattern mimicking an octopus trap [1,5]. Initial cases described postmenopausal women presenting with acute chest pain following emotional stress, and popularized terms such as ‘stress cardiomyopathy’ and ‘broken heart syndrome’ [1,5]. Subsequent observations showed TTS following a wide range of physical and neurological stressors that may even occur without an identifiable trigger, supporting the broader use of the term ‘Takotsubo syndrome’ in contemporary cardiology [2,4,6].

### 2.2. Core Diagnostic Elements

The diagnosis of TTS is based on characteristic, but non-specific, criteria [2,3,4]. Contemporary consensus documents emphasize the core component of transient regional wall motion abnormalities involving more than one part of the coronary distribution, without evidence of critical stenosis on angiography [1,2]. Troponin release is usually modest, often out of proportion to the severity of systolic dysfunction and is accompanied by large increases in natriuretic peptides [4,12]. Various electrocardiogram abnormalities, such as ST-segment elevation, ST-segment depression, extensive T-wave inversions, and QT prolongation, are very common and non-specific [4,12]. Cardiac MRI can be essential in making a diagnosis by showing evidence of myocardial edema without significant late gadolinium uptake, thus distinguishing between TTS, coronary infarction, and myocarditis [17,18]. The return of ventricular dysfunction within days to weeks is anticipated, which aids in diagnosis [1,4] (Table 1).

### 2.3. InterTAK Diagnostic Criteria

The InterTAK Diagnostic Criteria are currently the most commonly accepted criteria for TTS [2,3]. These criteria recognize that TTS may coexist with coronary artery disease if the wall motion abnormality is disproportionately small compared to the coronary findings [2]. The most significant advancement within the InterTAK criteria is the distinction between cases of primary TTS, usually activated by stressful situations and identified in the community, and secondary TTS, occurring within the context of acute medical, surgical, or neurological diseases [2,3]. Physicians use the InterTAK Diagnostic Score as an instrument to approach the probability of TTS without coronary angiography through variables including female sex, emotional and physical triggers, neurological and psychological conditions, ECG abnormalities, and QTc interval prolongation [8]. Although the score enhances the sensitivity and specificity of TTS, invasive coronary studies are required because of its considerable overlap with acute coronary syndromes [1,2].

### 2.4. Anatomical Variants

Various patterns of contraction abnormalities in the left ventricle have been described in TTS, depending on the susceptibility and pathophysiology involved [2,4,19]. The most common phenotype is apical ballooning, followed by mid-ventricular, basal (reverse), focal, and global variants, each defined by a characteristic distribution of transient wall-motion abnormalities [1,2,4,19]. Right ventricular involvement occurs in approximately one-third of cases and is associated with worse hemodynamic compromise [20]. Detailed descriptions of individual variants are summarized in Table 2 and discussed further in Section 5.2.

Recent studies have increasingly confirmed that TTS comprises various mechanistically different phenotypes as opposed to being part of the same disease [4,7]. A catecholamine-predominant type, known as significant and intense adrenergic episodes, is mainly induced by psychological stress or exogenous catecholamines and usually manifests as apical ballooning [2,4,21]. A coronary microvascular dysfunction–predominant phenotype has been proposed as a contributor to atypical Takotsubo variants and is accompanied by impairment of coronary flow reserve and endothelial dysfunction [10,22]. The neurogenic type derives from acute brain damage following abrupt episodes of central autonomic dysfunction and, as often, insular damage [14,15,16]. In conclusion, the chronic susceptibility type has been proposed to exist within those in whom chronic abnormalities of bioenergetic, hormonal, autonomic, or microvascular components are present and thus are vulnerable to atypical variants of TTS without apparent pathogenic agents [4,7]. Detailed descriptions of individual variants are summarized in Table 2 and discussed further in Section 5.2.

## 3. Epidemiology and Clinical Triggers

### 3.1. Incidence and Global Epidemiology

TTS represents approximately 1–3% of all cases presenting with acute coronary syndrome, although the true rate is likely underestimated, especially in atypical forms and in critically ill patients who are not evaluated with coronary angiography [1,5,11]. This condition has been reported globally, with largely consistent demographic trends across different regions [1,6]. The increasing recognition over time is likely caused by improved access to cardiac imaging, greater clinical awareness, and wider identification of non-classic variants [11]. TTS is more frequently diagnosed in centers with cardiac MRI capabilities, suggesting that the global incidence figures may still be incomplete [3,17]. Nevertheless, it is now clear that TTS is a major cause of acute heart failure, particularly in older women [1,2]. Early descriptions framed Takotsubo syndrome as a rare, emotionally triggered condition; however, improvements in imaging availability have substantially broadened recognition, revealing TTS as a frequent cause of acute heart failure across diverse clinical contexts.

### 3.2. Demographic Characteristics

TTS primarily affects postmenopausal women, accounting for more than 80–90% of cases [1,2]. The typical age at presentation ranged from 60 to 75 years [1]. Estrogen deficiency is thought to increase vulnerability by promoting microvascular dysfunction, autonomic imbalance, and heightened sensitivity to catecholamines [4,5]. Although less common in men and younger individuals, these groups tend to experience more severe hemodynamic compromise and higher complication rates [1,23]. Ethnic variation is limited, although differences in variant patterns and triggering events have been noted across populations [1,2,5].

### 3.3. Clinical Triggers and Precipitating Factors

Takotsubo syndrome may be precipitated by a broad spectrum of emotional, physical, neurological, infectious, and pharmacologic stressors. Although early observational reports emphasized emotional precipitants, contemporary registries demonstrate that physical and neurological triggers now account for the majority of cases, particularly among hospitalized and critically ill patients [1,24]. Emotional stressors (e.g., bereavement, fear, anger, and positive emotional events) remain associated with primary TTS and classic apical ballooning, whereas physical stressors such as sepsis, respiratory failure, surgery, trauma, metabolic derangements, and catecholamine exposure predominate in contemporary cohorts and are more frequently linked to atypical variants and acute complications [1,2,24].

Neurological insults, including stroke, subarachnoid hemorrhage, seizures, and traumatic brain injury, represent particularly potent triggers and are associated with severe systolic dysfunction, basal or mid-ventricular patterns, and increased early mortality [2,14]. A substantial proportion of patients present without an identifiable trigger, suggesting underlying vulnerability related to autonomic dysregulation, endothelial or microvascular dysfunction, hormonal factors, or genetic predisposition [2,5]. During the COVID-19 era, both infection-related inflammatory injury and widespread psychosocial stress have been associated with increased incidence and greater clinical severity of TTS [12,25]. Trigger-specific phenotypes, clinical severity, and short-term prognosis are summarized in Table 3.

## 4. Pathophysiology

The pathophysiology of Takotsubo syndrome is composed of mechanisms supported by varying strengths of clinical evidence. For instance, myocardial stunning and autonomic dysregulation are supported by consistent clinical, biomarker, and translational data, whereas abnormalities in coronary microvascular function, myocardial energetics, calcium handling, and central nervous system connectivity are supported by a combination of imaging studies, invasive physiology, and smaller mechanistic cohorts. The distinction between established concepts and novel hypotheses are highlighted throughout this section.

### 4.1. Catecholamine Surge and Sympathetic Overdrive

A key mechanism of TTS is a sudden surge in catecholamines, which leads to direct myocardial toxicity and transient ventricular dysfunction. Research has shown that catecholamine levels in patients with TTS are often significantly higher than those in patients with acute myocardial infarction [26]. Sustained β-adrenergic stimulation can cause a shift in β2-receptor signaling from Gs to Gi protein coupling, resulting in negative inotropic effects, especially in the apical myocardium, where β-receptor density is higher [21]. This catecholamine overload causes calcium imbalance, oxidative damage, mitochondrial dysfunction, and contraction-band necrosis, which together drive the reversible systolic dysfunction observed in TTS [4,26]. The frequent links between TTS and emotional stress, catecholamine use, and pheochromocytoma support the central role of sympathetic overactivation in disease onset [2,7].

### 4.2. Coronary Microvascular Dysfunction and Vasospasm

Coronary microvascular dysfunction (CMD) is increasingly being acknowledged as a critical factor in TTS. Many patients exhibit impaired coronary flow reserve, abnormal microvascular resistance despite the absence of obstructive coronary lesions, and some demonstrate inducible coronary vasospasm on acetylcholine testing [10,22]. PET imaging shows that the coronary flow reserve can be temporarily reduced during acute episodes, emphasizing dynamic microvascular involvement [27]. CMD is thought to arise from catecholamine-induced damage to the endothelium, heightened vascular reactivity, and impaired nitric oxide signaling, particularly in postmenopausal women with reduced endothelial reserve [4,8]. Microvascular impairment can then increase myocardial metabolic stress, contributing to regional susceptibility, accounting for the patterns in atypical and non-apical variants [9,10,22]. Importantly, recent pooled analysis have shown that impaired microvascular parameters are now each independently associated with an increased incidence of in-hospital complications, delayed left ventricular function recovery, and poorer outcomes in patients with TTS, thus confirming the prognostic significance of CMD rather than a purely mechanistic observation [28]. Persistent endothelial dysfunction may also play a role in symptom onset and recurrence [29].

### 4.3. Myocardial Energetics, Calcium Handling, and Contraction-Band Necrosis

In addition to temporary systolic dysfunction, TTS involves the significant disruption of energy metabolism and calcium regulation in the myocardium [9]. Imaging studies have shown decreased phosphocreatine-to-ATP ratios and reduced energy reserves, which can persist even after the ejection fraction normalizes [9]. Catecholamine-induced mitochondrial injury leads to oxidative stress, calcium overload, and metabolic instability [4,30]. Histological examination revealed contraction-band necrosis, a defining feature of catecholamine-related injury, which further links adrenergic stress to acute cardiac stunning [26]. These metabolic and structural changes likely explain lingering fatigue and exercise intolerance during recovery [9,29].

### 4.4. Left Ventricular Geometry, Regional Susceptibility, and Variants

The heterogeneous ventricular involvement observed in TTS is thought to reflect regional differences in left ventricular geometry, wall stress distribution, sympathetic innervation, and β-adrenergic receptor density rather than distinct anatomic disease entities [4,21]. Segments exposed to higher wall stress and greater adrenergic signaling appear more vulnerable to catecholamine-mediated myocardial stunning, while concomitant coronary microvascular dysfunction may further modulate regional susceptibility [4,7].

### 4.5. Sex Hormones, Genetics, and Susceptibility

The high incidence of TTS in postmenopausal women indicates a significant hormonal influence. Estrogen helps regulate endothelial function, autonomic balance, and β-adrenergic signaling; therefore, its absence may intensify catecholamine toxicity and weaken microvascular protection [5,8]. Genetic predisposition is also under investigation, with some studies linking TTS to variants in adrenergic receptors, G-protein-coupled receptor kinases, and stress-related pathways, although no single gene has been identified as a causative [31]. Hormonal and genetic factors likely interact with environmental triggers, lowering the threshold for TTS development and increasing the likelihood of recurrence [5,31].

### 4.6. Brain–Heart Axis and Central Autonomic Network

The brain–heart axis is increasingly recognized as the primary contributor to TTS, supported by functional neuroimaging, observational cohort studies, and translational models. Neuroimaging reveals structural and functional abnormalities in key areas responsible for autonomic and emotional regulation, including the amygdala, insular cortex, anterior cingulate cortex, and brainstem [15,32,33]. Elevated resting activity in the amygdala has been linked to increased sympathetic drive and a higher risk of TTS, suggesting that chronic stress-related brain changes may precede cardiac events [34]. Functional MRI has shown lasting disruptions in the connections between the limbic and autonomic systems, even after apparent heart recovery, reinforcing the concept of long-term autonomic dysregulation [32,33]. These findings explain the frequent overlap between neurological disorders and TTS.

### 4.7. Chronic Remodeling and Persistent Subclinical Dysfunction

Although TTS is typically labeled reversible, evidence shows that many patients continue to experience structural and functional heart changes after their ejection fraction returns to normal [29,35]. Speckle-tracking echocardiography revealed persistent reductions in both global and regional strains, suggesting ongoing subtle systolic dysfunction [29,35]. Other studies have highlighted continued microvascular impairment and endothelial dysfunction during long-term follow-up [22,29]. These lingering abnormalities are likely to contribute to symptoms such as fatigue and limited exercise capacity [9,29]. Outcomes, including hospitalization for HF and mortality, are similar to those after myocardial infarction [23,36]. Recurrence, observed in 4–10% of patients, further supports the idea of chronic vulnerability rather than full recovery [37,38].

### 4.8. Integrated Multimodal Model of Takotsubo Syndrome Pathophysiology

**Figure 1:**
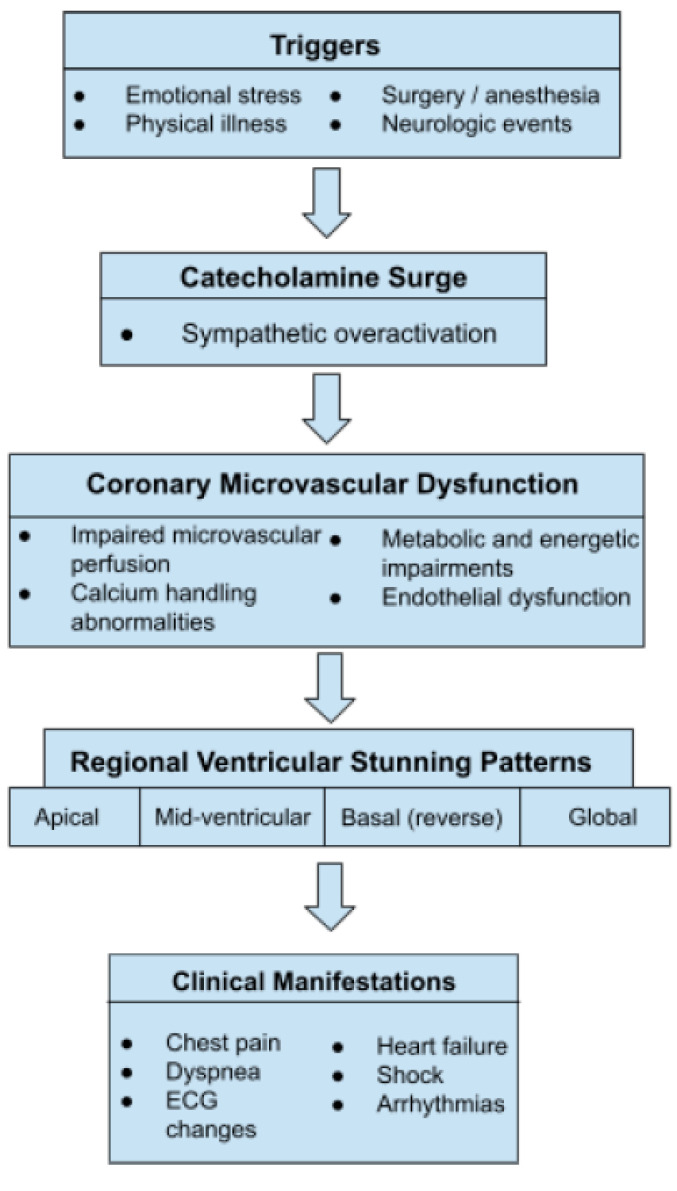
Multimodal pathophysiology model of Takotsubo syndrome. Acute emotional, physical, or neurologic stressors trigger sympathetic overactivation and catecholamine excess, leading to myocardial stunning, coronary microvascular dysfunction, and metabolic impairment. Interactions among neurohumoral, microvascular, and myocardial mechanisms result in transient ventricular systolic dysfunction and characteristic clinical manifestations.

TTS is best understood as the result of converging neurohumoral, myocardial, and microvascular mechanisms rather than a single pathogenic process. Acute sympathetic activation interacts with regional myocardial susceptibility, coronary microvascular dysfunction, and metabolic impairment to produce transient ventricular systolic dysfunction. These mechanisms act in a dynamic and interdependent manner, accounting for the heterogeneity of clinical presentation, ventricular involvement, and recovery trajectories observed across patients. This integrated framework provides a unifying explanation linking diverse triggers to a shared final phenotype of reversible myocardial dysfunction. Clinical manifestations represent the downstream effects of the neurocardiac, microvascular, and metabolic interface, as summarized schematically in Figure 1.

### 4.9. Strength of Evidence Across Proposed Pathophysiologic Mechanisms

Of these mechanisms, those most strongly supported clinical and experimental evidence in biomarker studies, imaging data, and observations in pheochromocytoma and acute neurologic injury include catecholamine-induced myocardial stunning and central autonomic dysregulation. Although definitive cause remains incompletely established, coronary microvascular dysfunction is supported by invasive coronary physiology, myocardial perfusion imaging, and endothelial function studies; while abnormalities in myocardial energetics, calcium handling, and metabolic remodeling are primarily supported by translational studies and small mechanistic cohorts. The relative contribution of each pathway may differ depending on the nature of the initiating trigger and the susceptibility of the affected individual [4,9,10,21,22,26].

## 5. Clinical Presentation and Variants

### 5.1. Typical Acute Presentation

TTS typically presents in a manner that closely resembles acute coronary syndrome, with patients experiencing sudden chest pain, shortness of breath, or near syncope after a physical or emotional stressor [1,2]. Electrocardiographic changes are present in most cases, including ST-segment elevation, T-wave inversion, and significant QTc prolongation, which characteristically evolve over several days [39,40]. Blood tests usually reveal only mild elevations in troponin but disproportionately elevated natriuretic peptides, which help distinguish TTS from myocardial infarction [1,40]. Imaging often reveals transient wall motion abnormalities that extend beyond the area of a single coronary artery, with apical ballooning being the most common finding [1,2]. Although many patients are stable, up to one-third can develop complications such as low blood pressure, pulmonary edema, arrhythmias, or cardiogenic shock, especially in cases triggered by neurological or physical stress [1,24]. Prompt coronary angiography is essential for accurate diagnosis in the acute phase [2,3]. The clinical features of typical and atypical presentations are compared in Table 3.

### 5.2. Clinical Heterogeneity and Prognostic Implications

**Figure 2:**
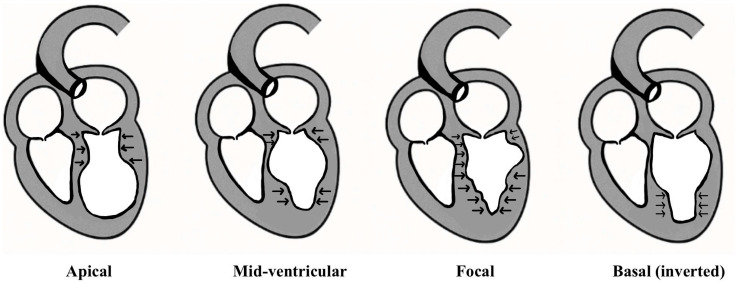
Schematic illustration of the principal left ventricular contraction patterns observed in Takotsubo syndrome. Arrows indicate segments demonstrating preserved or hyperdynamic systolic contraction.

Clinical expression of Takotsubo syndrome is heterogeneous and has important prognostic implications. Secondary and atypical presentations are less likely to manifest with chest pain or ST-segment elevation and more frequently present with acute heart failure, cardiogenic shock, or complications related to the underlying illness [12,19]. RV involvement is a key modifier of prognosis and is associated with greater hemodynamic instability, arrhythmias, thromboembolic risk, and prolonged hospitalization [20]. Early recognition of high-risk clinical patterns is essential for appropriate monitoring and supportive management. The anatomical distribution of ventricular dysfunction across Takotsubo variants is illustrated in Figure 2.

### 5.3. Triggers in Neurocritical and Perioperative Settings

Secondary TTS frequently occurs in hospitalized patients with significant physiological stress [24]. Neurologic events such as subarachnoid hemorrhage, ischemic stroke, and seizures activate central autonomic pathways and are closely associated with non-apical variants and severe hemodynamic compromise [14,24,41,42]. The perioperative period also poses a high risk, and factors such as anesthesia, blood loss, hypoxia, and abrupt sympathetic activation can serve as triggers [43,44]. These secondary forms of TTS tend to be more severe than primary stress-related cases and are associated with a higher incidence of cardiogenic shock and life-threatening arrhythmias [24].

## 6. Diagnostic Evaluation and Differential Diagnosis

**Figure 3:**
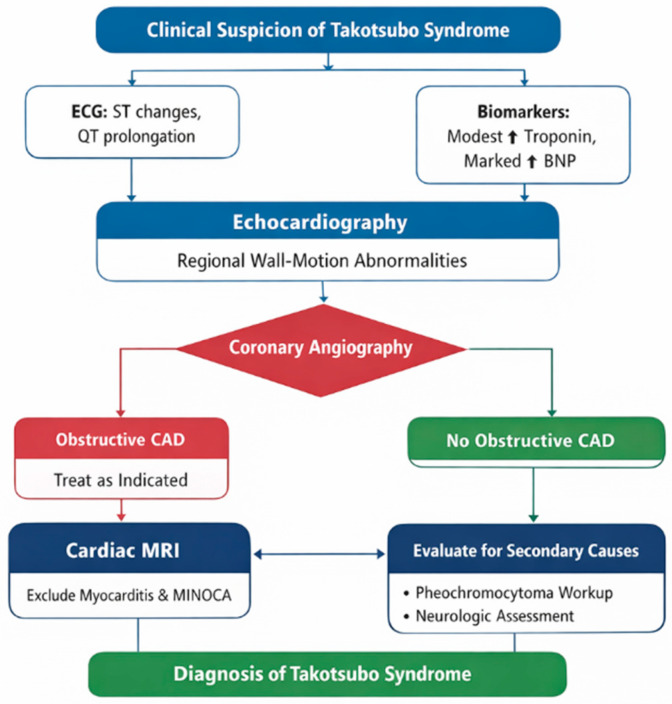
Diagnostic algorithm for Takotsubo syndrome. Flowchart illustrating a structured diagnostic approach incorporating clinical suspicion, ECG and biomarker findings, echocardiography, coronary angiography, cardiac MRI, and exclusion of alternative diagnoses.

Due to the considerable overlap between Takotsubo syndrome and other conditions that lead to acute myocardial injury, an organized diagnostic approach is necessary. The initial work-up is dependent upon clinical suspicion of presenting cardiac symptoms occurring in the context of emotional, physical, or neurological stressors. These stressors should be reflected by electrocardiographic abnormalities and a biomarker profile characterized by modest troponin elevation with abnormally elevated natriuretic peptides. Transthoracic echocardiography serves as the first-line imaging modality to identify transient regional wall-motion abnormalities extending beyond a single coronary territory. In most cases, there remains an urgent need for coronary angiography for the exclusion of obstructive coronary artery disease. For patients without coronary disease, cardiac magnetic resonance imaging helps to distinguish between TTS from myocarditis and myocardial infarction with non-obstructive coronary arteries (MINOCA). Assessments for secondary causes of MINOCA, such as pheochromocytoma or acute neurological damage, should additionally be considered in appropriate clinical contexts. This stepwise diagnostic framework is summarized in Figure 3, with established diagnostic tools used in routine clinical practice distinguished from adjunctive techniques that remain under evaluation.

### 6.1. InterTAK Diagnostic Criteria and Score

Diagnostic confirmation of TTS is based on the InterTAK Diagnostic Criteria, which integrate characteristic transient ventricular wall-motion abnormalities extending beyond a single coronary territory, modest biomarker elevation, and exclusion of alternative causes (see Section 2.3) [2,3,4]. The InterTAK Diagnostic Score may assist in estimating the pre-test probability of TTS in selected clinical contexts; however, due to substantial overlap with acute coronary syndromes, invasive coronary angiography remains mandatory in the acute setting [2,3].

### 6.2. ECG and Biomarker Profiles

ECG abnormalities are present in most cases and range from ST elevation and depression in early presentations through extensive T-wave inversions, and onwards into predominant QTc intervals in the subacute phase [2,39]. The biomarker profile is characterized by mild and moderate elevations in troponin levels and disproportionate increases in natriuretic peptides [40,45]. The ECG-biomarker mismatch may support discrimination between TTS and other causes of acute myocardial injury; however, it lacks sufficient specificity as a standalone diagnostic tool and should be regarded as an adjunct rather than a definitive test [1,26,39,40].

### 6.3. Echocardiography and Ventriculography

**Figure 4:**
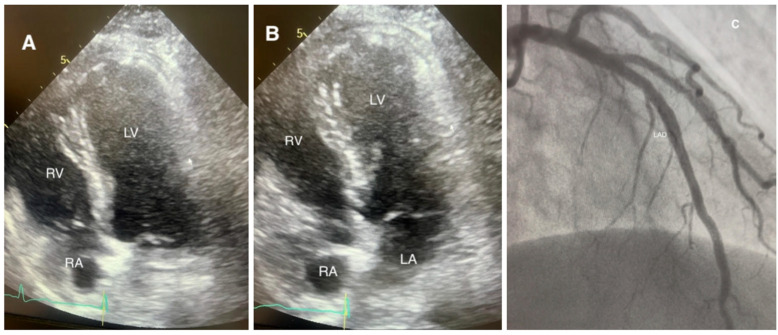
Apical four-chamber echocardiographic views of a female patient with Takotsubo syndrome following an incident of domestic abuse, admitted with chest pain and elevated cardiac enzymes. (**A**) Left ventricle during diastole. (**B**) Left ventricle during systole with akinetic and ballooned LV apex. (**C**) Coronary angiogram demonstrating patent coronary arteries. RV = right ventricle; RA = right atrium; LV = left ventricle; LA = left atrium; LAD = left anterior descending artery. (Courtesy of Dr. Raed Bargout).

Transthoracic echocardiography is the primary imaging technique used to diagnose various patterns, including apical ballooning and mid-, basal-, and focal variants [1,6,19]. Other features may include dynamic left ventricular outflow tract obstructions during systole, systolic anterior motion of the mitral valve, varying degrees of mitral regurgitation, and right ventricular abnormalities, whose presence indicates a poorer hemodynamic status and potential hemodynamic instability and shock [4,6,20,43]. Speckle-tracking echocardiography, while not yet incorporated into formal diagnostic criteria, typically shows sustained global longitudinal strain abnormalities that persist even after improvements in the left ventricular ejection fraction, suggesting subclinical dysfunction [9,35]. Ventriculography, as a part of coronary angiography, is still very sensitive for the evaluation of characteristic and atypical wall motion abnormalities, particularly in patients with suboptimal echocardiographic views [6,19]. Representative apical four-chamber echocardiographic views illustrating diastole and systolic apical ballooning in a classic case are shown in Figure 4A,B, together with the corresponding coronary angiogram demonstrating unobstructed coronary arteries (Figure 4C).

### 6.4. Cardiac MRI

Definitive differentiation between TTS, myocardial infarction, and myocarditis can often be achieved using cardiac MRI (CMR) [17]. On CMR, the common feature is regional myocardial edema on T2-weighted or T2 mapping in the absence of LGE, thereby helping distinguish TTS from infarction and inflammatory cardiomyopathy [4,17,18]. Additionally, CMR helps recognize atypical variants and evaluate right-sided involvement [4,6,17]. In certain cases, there is evidence of chronic inflammation and delayed recovery on follow-up CMR, thereby detecting abnormalities at the tissue level [9]. However, the diagnostic yield of CMR depends on early timing and availability, as myocardial edema may resolve on delayed imaging.

### 6.5. Coronary Angiography and Intravascular Imaging

Given its acute clinical presentation, emergency coronary angiography is often necessary [1,4,6]. Normal coronary arteries or non-critically significant plaques are often identified, which are neither evident nor plausibly accountable for wall motion defects [1,4,6]. When coronary artery disease is concurrent, intravascular ultrasound studies and optical coherence tomography may assist in ruling out conditions such as thrombi, emboli, ruptured plaques, and spontaneous coronary dissections, which are all parts and parcels of the diagnosis of myocardial infarction with non-obstructive coronary artery disease (MINOCA) [46,47]. The characteristic ballooning patterns of TTS on left ventriculography during coronary angiography are still useful [6,19].

### 6.6. Limitations of Diagnostic Modalities

Each diagnostic modality used in TTS has important limitations. Although transthoracic echocardiography is rapid and widely available, it may underestimate focal or subtle variants of TTS. Additionally, coronary angiography is unable to assess microvascular dysfunction and myocardial inflammation. While cardiac MRI is able to provide critical tissue-level differentiation between TTS, myocarditis, and myocardial infarction, its usage is limited by patient instability, contraindications, and high cost. Myocardial edema has its optimal sensitivity for detection in the first 3–7 days following presentation, but can resolve on delayed imaging studies. Biomarkers lack disease specificity and must be interpreted within the broader clinical and imaging context.

### 6.7. Differential Diagnosis: Pheochromocytoma, Myocarditis, MINOCA, and Sepsis-Induced Cardiomyopathy

The differential diagnoses of TTS are numerous and often consist of conditions with similar presentations. Other conditions, such as acute coronary syndrome, must be ruled out by urgent angiography, as per the guidelines [3,4,6]. Other diagnoses, namely myocarditis, may present with chest pain, ECG abnormalities, and elevated troponin levels, but typically show patchy or epicardial LGE patterns on CMR according to the updated Lake Louise Criteria [18]. Pheochromocytoma crisis may simulate TTS, featuring intense sympathetic stimulation and identical patterns of wall motion abnormalities; hence, biochemical evaluation should be considered in cases of recurrent episodes of adrenergic stimulation [4,6,47]. MINOCA is used as an overarching term for non-obstructive coronary artery occlusions secondary to plaque rupture, vasospasm, embolism, dissection, and myocarditis and can only be differentiated by CMR and OCT/IVUS studies, reflecting the clinical scenario [3,46].

Takotsubo syndrome in critically ill patients remains largely underdiagnosed and can be complicated with sepsis-induced cardiomyopathy (SIC) when invasive coronary angiography is not an option. Both can cause acute biventricular systolic dysfunction with elevated levels of natriuretic peptides, mild troponin elevation, and instability in a setting of systemic inflammation. However, SIC will have a global and reversible pattern of myocardial dysfunction without abnormality in ventricular geometry, whilst TTS will have regional wall motion abnormalities extending beyond a single vascular territory. Cardiac MRI can help if available, with a characteristic pattern of myocardial edema without late gadolinium enhancement for TTS, but none for SIC. Although overlap is likely present, a role for inflammatory stress in this setting can potentially be a precipitant rather than a surrogate alternative.

## 7. Risk Stratification and Prognosis

### 7.1. Acute Complications and In-Hospital Outcomes

Although TTS is often perceived to be reversible, early risk stratification is essential given the substantial risk of acute complications and in-hospital mortality, comparable to those observed in acute coronary syndromes [1,4,6]. Up to one-third of patients may experience hemodynamic instability, including hypotension, acute heart failure, and cardiogenic shock, requiring vasopressors or mechanical support [1,48,49]. Arrhythmias are frequent, with presentations including ventricular tachyarrhythmias, atrial fibrillation, and torsades de pointes, typically linked to significant QTc prolongation [1,4,6]. Patients with right ventricular dysfunction, dynamic obstruction of the left ventricular outflow tract, and moderate-to-severe mitral regurgitation are at a heightened risk of acute decompensation [4,6,20]. Contemporary registry data indicate in-hospital mortality rates of 2–5%, with substantially worse outcomes in secondary TTS associated with physical or neurologic stressors [1,4,24]. Several studies report a higher incidence of acute complications and in-hospital mortality in men; however, analyses adjusting for cardiogenic shock severity suggest that shock stage, rather than sex, is the dominant determinant of survival in advanced disease [50].

### 7.2. InterTAK Prognostic Score

The InterTAK Prognostic Score was developed to stratify short-term risk following initial stabilization in patients with TTS [4]. This score integrates clinical variables including age, LV ejection fraction, presence of physical or neurological triggers, atrial fibrillation, and right ventricular involvement to predict acute complications, cardiogenic shock, and mortality [51]. Patients presenting with secondary TTS, particularly those associated with neurological or critical illness, consistently demonstrate higher prognostic scores and increased requirements for intensive care, mechanical circulatory support, and prolonged hospitalization [1,24,51]. Observational analyses further suggest that higher SCAI shock stages may be a stronger determinant of in-hospital outcomes than trigger category alone, supporting incorporation of shock-based risk stratification tools in high-risk patients [4,51].

### 7.3. Long-Term Morbidity, Mortality, and Recurrence

From a prognostic standpoint, long-term outcomes in TTS are strongly influenced by early risk profile and recovery trajectory. Although the left ventricular ejection fraction typically recovers within a few days to weeks, long-term recovery is often incomplete [9,29,35]. Large registry studies have shown that the 5-year mortality rates in TTS are comparable to those seen after acute myocardial infarction, with many deaths attributable to non-cardiac comorbidities such as cancer or neurologic conditions [1,25,38]. Ongoing symptoms, including fatigue, reduced exercise tolerance, shortness of breath, and emotional distress, are common and often reflect persistent myocardial strain, CMD, and energetic abnormalities [9,29,35]. Recurrence occurs in approximately 4–10% of patients and often presents with a different anatomical variant, suggesting that the condition reflects an underlying chronic vulnerability rather than an isolated acute event [1,4,37,38]. Reported recurrence rates vary across studies due to differences in follow-up duration, diagnostic criteria, patient populations, and ascertainment methods, highlighting significant heterogeneity in the available data. Individuals with ongoing physical illnesses, psychiatric disorders, or chronic emotional stress are at particularly high risk for recurrence [1,4,37]. Additionally, patients with delayed normalization of ventricular function experience higher long-term mortality, increased heart failure hospitalization, and persistent symptoms compared with those demonstrating early recovery [52] (Table 4).

## 8. Management

It is important to note that the management of Takotsubo syndrome is largely guided by observational data, registry analyses, and expert consensus rather than robust randomized controlled trials. Due to the acute, heterogeneous, and typically reversible nature of the syndrome, most therapeutic strategies stem from the framework of heart failure and cardiogenic shock management, while evidence supporting specific interventions remains limited. Current recommendations emphasize individualized, phenotype-directed management rather than standardized treatment algorithms.

### 8.1. Acute Hemodynamic Stabilization

Acute management in Takotsubo syndrome centers on early hemodynamic assessment, identification of left ventricular outflow tract obstruction (LVOTO), as well as avoidance of interventions that may exacerbate dynamic obstruction or sympathetic stress. In LVOTO-positive patients, therapies that increase contractility may exacerbate obstruction, whereas management prioritizes cautious volume resuscitation and afterload augmentation with pure vasoconstrictors such as phenylephrine [4,6,43,44]. However, more recent data indicate that clinically significant LVOTO worsening with vasoactive or inotropic support is relatively uncommon, even among patients treated for cardiogenic shock [50], likely reflecting careful preload optimization, hemodynamic context, and phenotypic variability across TTS presentations, and concern for obstruction should not delay life-saving hemodynamic support when clinically indicated. In contrast, LVOTO-negative patients with severe systolic dysfunction may require judicious inotropic or temporary circulatory support [4,6,49]. Acute pulmonary edema or respiratory failure may necessitate non-invasive or invasive ventilation [1,4,6]. As patients with secondary TTS, particularly those triggered by neurological or critical illnesses, are at the greatest risk of early instability, monitoring in an intensive care environment is often recommended [1,4,24]. These recommendations are based primarily on registry data and expert consensus, as no randomized trials have evaluated acute vasoactive strategies specifically in Takotsubo syndrome.

### 8.2. Heart Failure-Directed Therapy

Blocking neurohormonal activation is a central strategy during both the acute and early recovery phases [4]. ACE inhibitors or ARBs are commonly prescribed and may help improve long-term survival and prevent adverse remodeling [51]. Outcome-driven analyses from contemporary cohorts suggest an association between ACE inhibitor or angiotensin receptor blocker therapy and improved survival in Takotsubo syndrome, particularly among patients with more severe left ventricular dysfunction or higher comorbidity burden [51]. Beta-blockers are used to reduce the adrenergic drive, and recent large observational studies suggest a survival benefit in those with physical trigger-induced TTS or secondary TTS; although their effectiveness in preventing recurrence remains controversial [1,4,37]. Apart from their role in adrenergic modulation, beta-blockers have proven utility in other TTS pathophysiology-related pathways, such as autonomic imbalance modulation, calcium handling, myocardial oxygen demand reduction, and suppression of inflammatory responses linked to stress. Diuretics are appropriate for managing fluid overload, whereas mineralocorticoid receptor antagonists may be considered for patients with more pronounced left ventricular dysfunction [4,6]. Medications may be tapered as function improves; however, ongoing treatment may be warranted for patients with persistent strain abnormalities or recurrent episodes [4,6,9,35,37]. Evidence supporting neurohormonal blockade in Takotsubo syndrome is derived largely from observational cohorts, and randomized trials demonstrating definitive benefit are lacking.

### 8.3. Arrhythmia and Thromboembolic Risk Management

Arrhythmias, including atrial fibrillation, ventricular tachyarrhythmias, and torsades de pointes, pose significant early risks. Continuous ECG monitoring is recommended in patients with prolonged QT intervals, low ejection fractions, or a history of fainting [4,6,39]. Left ventricular thrombi can form in 2–8% of Takotsubo patients, with a higher risk in patients with marked apical akinesia, substantial reduction in ejection fraction, or delayed ventricular recovery. Management of ventricular arrhythmias includes magnesium replacement, aggressive correction of electrolyte imbalances, and avoidance of QT-prolonging medications [4,6,39]. Anticoagulation should be initiated in patients with significant apical akinesis, documented left ventricular thrombus, or severely reduced ejection fraction and continued until wall motion normalizes [4,6,48]. Anticoagulation strategies in Takotsubo syndrome are similarly informed by observational data from other cardiomyopathy populations. Registry-based analyses have identified severe apical ballooning, left ventricular ejection fraction ≤ 30–35%, extensive akinetic segments, delayed recovery of ventricular function, and elevated inflammatory or coagulation markers as independent predictors of left ventricular thrombus formation, allowing early identification of patients who may benefit from intensified imaging surveillance and prophylactic anticoagulation [1,48,51].

### 8.4. Mechanical Circulatory Support in Cardiogenic Shock

Mechanical circulatory support (MCS) can be lifesaving in patients with persistent cardiogenic shock [49]. The choice of the device depends on the presence of LVOTO. In patients with LVOTO, intra-aortic balloon pumps are avoided, as they may worsen obstruction; instead, VA-ECMO or ECMELLA (combined ECMO and Impella) may be used for cases involving biventricular failure or deep shock [4,6,49]. In patients without LVOTO, percutaneous devices such as Impella or TandemHeart may help improve forward flow and reduce wall stress [4,6,49]. MCS is generally a temporary measure, as most patients recover their ventricular function after the acute phase [1,49]. Data supporting the use of mechanical circulatory support in Takotsubo-related cardiogenic shock are limited to case series and registry analyses, and device selection is guided by physiologic principles rather than trial-based evidence.

### 8.5. Chronic Management and Secondary Prevention

There are no current randomized controlled trials evaluating pharmacologic strategies for recurrence prevention in Takotsubo syndrome. Long-term treatment focuses on preventing recurrence and managing persistent symptoms. Beta-blockers may help patients with heightened adrenergic responses or arrhythmia risk, although data on the prevention of recurrence are inconclusive [1,4,37]. The continued use of ACE inhibitors or ARBs may be beneficial, especially in patients with ongoing functional impairments or hypertension [14,51]. Psychological stress, psychiatric disorders, and chronic physical illnesses should be addressed because of their strong association with recurrence and chronic morbidity [1,4,37,47]. Follow-up imaging, including strain studies, may help identify patients requiring extended therapy [9,35].

### 8.6. Rehabilitation, CBT, and Exercise: Emerging RCT Data

Emerging evidence from observational studies and early randomized trials supports the use of structured rehabilitation programs to aid recovery and improve the psychological health of patients with TTS [53]. Observational studies and early randomized trials have shown that guided aerobic and resistance training combined with comprehensive cardiac rehabilitation can enhance exercise tolerance, restore autonomic function, and improve quality of life [53,54]. CBT may also reduce anxiety, stress-related reactivity, and emotional triggers, particularly in patients with primary or recurrent TTS [53]. Although larger trials are needed, the current evidence supports the use of structured exercises and psychological care in long-term treatment plans for appropriate patients. Overall, the absence of large randomized controlled trials represents a major limitation in the evidence base for Takotsubo syndrome management and highlights an urgent need for prospective, outcome-driven studies.

## 9. Special Populations and Clinical Contexts

### 9.1. Neurogenic TTS (Stroke, SAH, Seizures)

Neurogenic TTS is known to be one of the most ‘toxic’ forms. It is usually induced by acute cerebrovascular and neurologic disturbances, which may include subarachnoid hemorrhage, ischemic stroke, intracerebral hemorrhage, generalized seizure activity, traumatic brain injury, and dysfunction of the central autonomic network [41,42,55]. Neurologic injury is a well-recognized precipitant of TTS, with mechanisms involving central autonomic dysregulation described in detail in Section 3 and Section 4. TTS and related neurogenic stress cardiomyopathy may complicate a substantial proportion of aneurysmal subarachnoid hemorrhage cases and often exhibit non-apical patterns of ventricular dysfunction, supporting the concept of regionally heterogeneous sympathetic activation [42,55]. In contrast to the emotional variant, neurogenic cases are much more severe, manifested by extensive systolic dysfunction and greater frequencies of cardiac arrest, multi-organ failure, arrhythmias, and in-hospital mortality [1,24,39]. There is often a significant delay in diagnosis because cardiac dysfunction is overshadowed by neurologic instability, thereby strongly emphasizing routine cardiac evaluation secondary to ECG, biomarkers, and echocardiography in cases of severe neurologic emergencies, as well as for any perturbations of cardiovascular disease [41,42,55].

### 9.2. Oncologic and Perioperative TTS

Takotsubo syndrome in oncologic and perioperative settings most commonly occurs as secondary TTS in the context of systemic illness, surgical stress, or exposure to cardiotoxic therapies, with underlying mechanisms discussed in Section 4. Several chemotherapies and targeted therapies, such as 5-fluorouracil, capecitabine, tyrosine kinase inhibitors, and immune checkpoint blockers, have been identified as potential inducers of endothelial dysfunction, vasospasm, and direct catecholaminergic toxicity [31,43,54]. The presence of cachexia, anemia, infections, and inflammation secondary to cancer increases the vulnerability [54]. Perioperative forms occur secondary to the induction of anesthesia, pain, hemodynamic instability, stimulation of airways, or surgical stress and may occur intraoperatively as abrupt episodes of hypotension, arrhythmias, and unexpected wall motion abnormalities on transesophageal echocardiography [43,44]. The outcome in the setting of cancers and surgery is less optimal than that in other instances of TTS, as antecedent conditions of systemic disease, immune compromise, and metabolic stresses often accompany secondary triggers [1,24,43,54].

### 9.3. COVID-19 and Other Systemic Inflammatory States

The coronavirus disease (COVID-19) pandemic has highlighted COVID-19 as a clinically important trigger context for secondary TTS. However, the incidence of TTS substantially increased in the early phase of the pandemic among infected individuals and those under intense psychosocial stress [25]. Proposed inflammatory and autonomic mechanisms are discussed in Section 3 and Section 4; clinically, COVID-associated TTS is notable for greater severity and a higher burden of cardiopulmonary complications [25]. The incidence of COVID-related TTS is often complicated by respiratory failure, high levels of troponin and BNP, and both right and left ventricular dysfunction, and is associated with high rates of shock and mortality compared to non-COVID TTS [25]. Similar secondary presentations have been reported in other severe systemic inflammatory states (e.g., sepsis, severe burns, pancreatitis, autoimmune exacerbations), where TTS is associated with higher illness severity and worse short-term outcomes [4,12,31].

### 9.4. Pediatric Takotsubo Syndrome

Although TTS has classically been thought of from an adult perspective, Takotsubo syndrome is increasingly being identified in children and adolescents. The largest series that has been published to date confirms a different triggering etiology in children with TTS, with neurologic insult such as seizures, intracranial hemorrhage, and traumatic brain injury being identified as the main precipitating factor in contrast to emotional distress in adults, which is most common in TTS patients [56]. Although there is increased acute illness in children at admission, prognosis and eventual return of ventricular function appear to be no different than in adults. Mechanistic considerations in pediatric TTS are presumed to overlap with adult forms and are not yet well characterized.

## 10. Future Directions

### 10.1. Biomarkers and Imaging-Based Phenotyping

Despite improvements in the diagnostic criteria, one of the challenges in diagnosing TTS is the lack of non-invasive biomarkers to differentiate it from acute coronary syndrome on presentation. Troponin and natriuretic peptides provide non-specific corroborating data, but the serial biomarker profile is virtually indistinguishable from that of other cardiac diseases [38,45]. More innovative approaches, such as circulating catecholamine metabolites, endothelial dysfunction biomarkers, cytokines, microRNAs, and metabolomic patterns, appear promising but are still inadequately validated [31,45]. Furthermore, non-invasive imaging techniques can define wall motion abnormalities, but they inadequately define the mechanism of susceptibility, as well as microvascular damage, in the affected walls [4,10,13,17]. Modern MRI approaches, such as parametric, strain, and stress-perfusion studies, may potentially allow for a more refined categorization, whereas nuclear and coronary flow reserve studies may provide better insights into microvascular damage [13,17,22]. A major limitation of current biomarker and imaging approaches is the lack of standardization across centers, particularly with respect to cardiac magnetic resonance protocols, timing of acquisition, and quantitative thresholds for edema, strain, and perfusion abnormalities. Additionally, most biomarkers have been evaluated in small, heterogeneous cohorts, which limit reproducibility and external validity. Without standardized validation frameworks, translation of these tools into routine clinical practice remains premature.

### 10.2. AI and Machine Learning in Diagnosis and Risk Prediction

Artificial intelligence and machine learning have the potential to augment future management by improving early diagnosis, classification, and risk prediction. The use of machine learning algorithms on ECG patterns, clinical profiles, echocardiographic variables, and cardiac MRI databases has shown promise in retrospective studies for distinguishing TTS from myocardial infarction and predicting hemodynamic instability, arrhythmic events, and outcomes [57,58]. The integration of large multicenter datasets, real-time telemetry, and generative models may enable prospective risk stratification in the future once adequately validated [57,58]. However, the lack of large, standardized, and annotated datasets, underrepresentation of atypical and secondary presentations, limited external validation, and absence of prospective implementation studies represent major barriers to routine clinical adoption as it raises concerns regarding algorithmic bias and generalizability [13].

### 10.3. Targeted Neurohumoral and Microvascular Therapies

The management of TTS remains mainly supportive, without any utility-proven disease-modifying agents [12,48]. This represents a major gap in our understanding of the mechanistic heterogeneity of TTS [13]. In catecholamine-driven variants, there may be utility in agents targeting sympathetic modulation, β-adrenergic pathway modulation, and stress axis modulation, whereas those with microvascular patterns may benefit from agents targeting vasodilation, endothelial stabilization, and anti-inflammatory regimens [4,10,21,31]. Experimental agents, such as endothelin receptor antagonists, rho-kinase inhibitors, central autonomic modulators, and metabolic agents, have shown a mechanistic rationale in model studies, but have yet to be tested in randomized controlled studies [4,31,59]. There is an urgent need for mechanism-based therapies that may correspond to the proposed pathophysiological patterns, namely catecholamine surge, microvascular dysfunction, neurogenic autonomic dysfunction, and chronic susceptibility patterns [7,8]. Translation of these mechanistic insights into effective therapies has been limited by phenotypic heterogeneity, transient disease course, ethical challenges in acute enrollment, and the absence of validated surrogate endpoints for therapeutic response.

### 10.4. Ongoing Clinical Trials

Several prospective and observational studies of Takotsubo syndrome are currently registered on ClinicalTrials.gov and EUdraCT, reflecting growing efforts to move beyond descriptive registries toward mechanism- and outcome-driven investigation. Ongoing studies include large observational registries characterizing clinical phenotypes and outcomes (NCT03663348), mechanistic investigations aimed at defining biological and autonomic heterogeneity (NCT04325321), and interventional trials evaluating pharmacologic strategies such as beta-blocker therapy (NCT06509074) and immunomodulatory approaches (NCT05946772). Additional registered studies are examining optimization of medical therapy and recovery trajectories (NCT04666454). Collectively, these trials aim to refine risk stratification, clarify disease mechanisms, and inform future guideline-directed management of Takotsubo syndrome.

### 10.5. Designing Future Clinical Trials

Progress in TTS is hampered by a lack of large prospective studies, non-uniform case definitions, and varying phenotypic classifications [13]. Moving forward, studies must address standardized diagnostic tools, such as the InterTAK Diagnostic Criteria and score, focus on both typical and atypical presentations, and be divided into primary and secondary cases [1,2]. Pragmatic trial designs should examine the use of therapies targeted at the phenotype, thereby interrogating the value of chronic pharmacologic approaches and then address managing high-risk groups, namely those secondary to neurogenic stimulation, right heart involvement, cardiogenic shock, and recurrent disease [4,13,48]. Follow-up is crucial for understanding longstanding functional capacity and arrhythmic, psychological, and recurrent disease [38,51,54]. Future randomized controlled trials should incorporate standardized entry criteria utilizing InterTAK Diagnostic Criteria, early imaging confirmation, and defined stratification based on trigger type and ventricular phenotype. Exclusion criteria should consist of active myocardial inflammation, significant obstruction of the coronary arteries, or other contributing factors of acute cardiomyopathy. Clinically meaningful primary endpoints should include in-hospital complications and restoration of ventricular function, while secondary endpoints should comprise assessments of arrhythmia burden, recurrence and quality of life.

## 11. Conclusions

TTS is a clinically important and heterogenous acute heart failure syndrome that extends beyond its traditionally perceived reversibility. Despite recovery of left ventricular systolic function in most patients, TTS is associated with substantial risks of in-hospital complications, persistent functional impairment, and recurrence, underscoring the importance of early recognition and vigilant physician action. Effective management necessitates phenotype-guided acute care, with particular attention to patients with secondary triggers, cardiogenic shock, or right ventricular involvement. Risk stratification should therefore prioritize hemodynamic severity and recovery trajectory rather than trigger category alone, to guide monitoring intensity and follow-up. Chronic management should integrate rehabilitation and supportive interventions aimed at addressing persistent symptoms as well as functional limitations. From a research perspective, advancing care beyond supportive management will depend on standardized phenotyping, prospective studies, and randomized trials targeting mechanism-driven therapies with clinically meaningful endpoints.

## Figures and Tables

**Table 1:** Clinical, Electrocardiographic, Biomarker, and Imaging Features of Takotsubo Syndrome.

Category	Typical Findings
Triggers	Emotional or physical stress; acute medical illness; surgery; adrenergic drugs; pheochromocytoma; chemotherapy agents.
Clinical Presentation	Acute chest pain; dyspnea; syncope; signs of acute heart failure; cardiogenic shock or arrhythmias in severe cases.
ECG	ST-segment elevation or depression; diffuse T-wave inversion; QTc prolongation in the subacute phase; dynamic changes over days to weeks.
Biomarkers	Mild–moderate troponin elevation; marked BNP/NT-proBNP elevation; disproportionate rise in natriuretic peptides relative to troponin.
Echocardiography	Regional wall-motion abnormalities not confined to a single coronary territory; apical, midventricular, basal, or focal patterns; reduced LVEF with early improvement.
CMR	Myocardial edema (T2/T2-mapping); absence of ischemic LGE; rare, faint, or atypical LGE reported in select cases; normalization of edema on follow-up imaging.
Coronary Angiography	No obstructive coronary disease; presence of non-critical plaques possible; no evidence of plaque rupture or thrombus.
Complications	LVOT obstruction; acute heart failure; arrhythmias; cardiogenic shock; apical thrombus; stroke; recurrence.

**Table 2:** Comparative overview of Takotsubo syndrome phenotypes, characteristic left ventricular wall-motion patterns, common triggering factors, electrocardiographic and biomarker features, and relative prognosis.

Takotsubo Phenotype	LV Wall-Motion Pattern	Typical Triggers	ECG/Biomarker Features	Prognosis
Apical (classic)	Apical akinesis or dyskinesis with basal hyperkinesis	Emotional stress (grief, fear, shock), acute psychological distress	ST-segment elevation or deep T-wave inversion; modest troponin elevation	Generally favorable; most common phenotype with high likelihood of full recovery
Mid-ventricular	Mid-LV akinesis with preserved apical and basal contraction	Physical stress, acute medical illness, neurologic events	ST-segment depression or non-specific changes; variable troponin elevation	Good recovery in most cases; recurrence risk similar to apical variant
Basal (reverse)	Basal akinesis with apical hyperkinesis	Catecholamine excess, pheochromocytoma, exogenous adrenergic agents, younger patients	Less frequent ST-elevation; relatively higher troponin levels	Higher risk of acute complications (LVOT obstruction, cardiogenic shock)
Focal	Isolated regional wall-motion abnormality	Physical stress, procedures, perioperative triggers	Often mimics focal myocardial infarction	Favorable prognosis; likely underdiagnosed due to subtle imaging findings
Global	Diffuse LV hypokinesis without regional predominance	Severe systemic illness, sepsis, shock, ICU-level stress	Marked biomarker elevation; diffuse ECG abnormalities	Worst short-term prognosis, higher rates of hemodynamic instability and mortality

**Table 3:** Integrated Comparison of Takotsubo Syndrome Trigger Categories, Ventricular Phenotypes, Clinical Features, and Prognosis.

Feature	Emotional/Typical TTS	Physical/Neurologic/Atypical TTS	TTS with RV Involvement
Typical trigger profile	Emotional stress (grief, fear, anger, positive emotional events)	Physical stress, neurologic disease, infection, malignancy, surgery, COVID-19	Severe physical or neurologic stress
Approximate proportion	~25–30%	~40–55%	~25–35%
Common LV phenotype	Apical (classic ballooning)	Mid-ventricular, basal (reverse), focal, or global	Often coexisting LV dysfunction
Chest pain	Common	Variable or absent	May be absent
Dyspnea	Common	Common	Prominent
ECG findings	ST-segment elevation, T-wave inversion, QT prolongation	Nonspecific or atypical changes	Sinus tachycardia, right-axis deviation
Biomarkers	Modest troponin elevation	Variable troponin elevation	Markedly elevated BNP
LV function	Transient regional dysfunction	Variable LV patterns	Frequently reduced
RV function	Preserved	Typically preserved	RV dilation and systolic dysfunction
Hemodynamic instability	Uncommon	More frequent	Common
Typical complications	Heart failure, arrhythmias	LVOT obstruction, cardiogenic shock	Acute RV failure, thromboembolism
Overall prognosis	Favorable	Intermediate	Worst short-term prognosis

**Table 4:** Acute and long-term mortality in Takotsubo syndrome and associated predictors.

Outcome Period	Reported Rates	Key Predictors	Notes
In-hospital mortality	~2–5%	Cardiogenic shock, RV involvement, physical/neurologic triggers, older age	Higher in secondary TTS
Short-term (30–90 days)	~4–8%	Shock severity, critical illness, comorbidities	Often non-cardiac deaths
Long-term mortality (5 years)	Comparable to MI	Cancer, neurologic disease, delayed LV recovery	Cardiac vs. non-cardiac causes vary
Recurrence	~4–10%	Psychiatric disease, chronic stress, physical illness	High inter-study variability

## Data Availability

Not applicable; no new data were created or analyzed in this study.

## Abbreviations

The following abbreviations are used in this manuscript:

ACS	Acute Coronary Syndrome
BNP	Brain Natriuretic Peptide
CBT	Cognitive Behavioral Therapy
CMD	Coronary Microvascular Dysfunction
CMR	Cardiac Magnetic Resonance Imaging
ECG	Electrocardiogram
HF	Heart Failure
ICU	Intensive Care Unit
IVUS	Intravascular Ultrasound
LGE	Late Gadolinium Enhancement
LVOTO	Left Ventricular Outflow Tract Obstruction
MCS	Mechanical Circulatory Support
MINOCA	Myocardial Infarction with Non-Obstructive Coronary Arteries
PET	Positron Emission Tomography
RCT	Randomized Controlled Trial
SAH	Subarachnoid Hemorrhage
TTS	Takotsubo Syndrome
VA-ECMO	Veno-Arterial Extracorporeal Membrane Oxygenation
